# Prediagnostic Serum Concentrations of Organochlorine Compounds and Risk of Testicular Germ Cell Tumors

**DOI:** 10.1289/ehp.0800359

**Published:** 2009-05-20

**Authors:** Mark P. Purdue, Lawrence S. Engel, Hilde Langseth, Larry L. Needham, Aage Andersen, Dana B. Barr, Aaron Blair, Nathaniel Rothman, Katherine A. McGlynn

**Affiliations:** 1 Division of Cancer Epidemiology and Genetics, National Cancer Institute, National Institutes of Health, Department of Health and Human Services, Bethesda, Maryland, USA; 2 Department of Epidemiology and Biostatistics, Memorial Sloan-Kettering Cancer Center, New York City, New York, USA; 3 The Norwegian Cancer Registry, Oslo, Norway; 4 National Center for Environmental Health, Centers for Disease Control and Prevention, Atlanta, Georgia, USA

**Keywords:** chlordanes, organochlorine compounds, polychlorinated biphenyls, *p,p*′-dichloro-diphenyldichloroethylene, testicular germ cell tumors

## Abstract

**Background:**

Recent findings suggest that exposure to organochlorine (OC) compounds, chlordanes and *p,p*′-dichlorodiphenyldichloroethylene (*p,p*′-DDE) in particular, may increase the risk of developing testicular germ cell tumors (TGCTs).

**Objective:**

To further investigate this question, we conducted a nested case–control study of TGCTs within the Norwegian Janus Serum Bank cohort.

**Methods:**

The study was conducted among individuals with serum collected between 1972 and 1978. TGCT cases diagnosed through 1999 (*n* = 49; 27–62 years of age at diagnosis) were identified through linkage to the Norwegian Cancer Registry. Controls (*n* =51) were matched to cases on region, blood draw year, and age at blood draw. Measurements of 11 OC insecticide compounds and 34 polychlorinated biphenyl (PCB) congeners were performed using gas chromatography/high-resolution mass spectrometry. Case–control comparisons of lipid-adjusted analyte concentrations were performed using the Wilcoxon signed-rank test. Odds ratios (ORs) and 95% confidence intervals (CIs) for tertiles of analyte concentration were calculated using conditional logistic regression.

**Results:**

TGCT cases had elevated concentrations of *p,p*′-DDE (tertile 3 vs. tertile 1 OR (OR_T3_) 2.2; 95% CI, 0.7–6.5; *p*_Wilcoxon_ = 0.07), oxychlordane (OR_T3_ 3.2; 95% CI, 0.6–16.8; *p*_Wilcoxon_ = 0.05), *trans*-nonachlor (OR_T3_ 2.6; 95% CI, 0.7–8.9; *p*_Wilcoxon_ = 0.07), and total chlordanes (OR_T3_ 2.0; 95% CI, 0.6–7.2; *p*_Wilcoxon_ = 0.048) compared with controls, although no ORs were statistically significant. Seminoma cases had significantly lower concentrations of PCB congeners 44, 49, and 52 and significantly higher concentrations of PCBs 99, 138, 153, 167, 183, and 195.

**Conclusions:**

Our study provides additional but qualified evidence supporting an association between exposures to *p,p*′-DDE and chlordane compounds, and possibly some PCB congeners, and TGCT risk.

The incidence of testicular germ cell tumors (TGCTs) has risen considerably in Caucasian populations for more than three decades and is now the most commonly diagnosed malignancy among men 15–34 years of age (Purdue et al. 2005). TGCTs can be grouped histologically into seminomas and nonseminomas. Seminomas are less aggressive than non-seminomas and peak in incidence at an older age (35–40 years vs. 25–30 years) (Moller 1993).

No adequate explanations for the remarkable increase in TGCT incidence have been identified, as the etiology of this cancer remains largely unknown. The only well-described risk factors are cryptorchidism, Caucasian ethnicity, and personal and family history of TGCT (McGlynn 2001). Epidemiologic findings suggest various factors that may influence TGCT development, including exposure to high maternal estrogen levels *in utero*, low androgen levels, mechanisms underlying infertility, and factors operating in peri natal life (McGlynn 2001). However, these hypotheses remain tentative.

The hypothesized role of sex hormones in testicular carcinogenesis has led to considerable interest as to whether exposure to environmental endocrine-disrupting chemicals such as organochlorine (OC) compounds may be associated with TGCT risk. OC compounds are synthetic chemicals used as pesticides and in various industrial applications until concerns over their extreme environmental persistence and possible health effects led many countries to ban their production and/or use in the 1970s and 1980s. Experimental findings suggesting that OC compounds exert weak estrogenic, anti estrogenic, and anti-androgenic effects (Bonefeld-Jorgensen et al. 2001; Danzo 1997; Kelce et al. 1995) have raised concern over their potential to influence the development of TGCTs and other male reproductive disorders speculated to be part of the testicular dysgenesis syndrome: cryptorchism, hypospadias, and impaired spermatogenesis (Skakkebaek et al. 2001; Toppari et al. 1996).

Three case–control studies have investigated the association between serum OC concentrations and TGCT risk to date. In a small hospital-based study (58 cases, 61 controls) conducted in Sweden, investigators found significantly higher serum concentrations of *cis*-nonachlor, a congener of the insecticide chlordane, among cases compared with controls (Hardell et al. 2003). In addition, mothers of cases were found to have higher serum levels of chlordane compounds (*cis*-nonachlor, *trans*-nonachlor, total chlordanes), the insecticide hexachlorobenzene (HCB), and poly-chlorinated biphenyls (PCBs)—chemicals used in a variety of industrial applications—than control mothers. The U.S. Servicemen’s Testicular Tumor Environmental and Endocrine Determinant (STEED) Study, a large case–control investigation of TGCTs (754 cases, 928 controls) conducted among U.S. servicemen, similarly found elevated pre-diagnostic concentrations of *cis*-nonachlor, *trans*-nonachlor, and the insecticide metabolite *p,p*′-dichlorodiphenyldichloroethylene (*p,p*′-DDE) among cases versus controls (McGlynn et al. 2008). However, a recent population-based study (246 cases, 630 controls) conducted in Washington State observed no evidence of association with OC compounds (Biggs et al. 2008). Findings from studies of cryptorchidism and hypospadias do not support an association with OC compounds (Bhatia et al. 2005; Damgaard et al. 2006; Flores-Luevano et al. 2003; Longnecker et al. 2002; Waliszewski et al. 2005).

We conducted a nested case–control within the Janus Serum Bank cohort of Norway, the country with the highest incidence of TGCTs in the world (Curado et al. 2007), to further investigate whether serum levels of 11 OC insecticide compounds and 34 PCB congeners are associated with risk of this cancer.

## Methods

### Study population

The Janus Serum Bank (The Cancer Registry of Norway, Oslo, Norway) was established in 1972 and consists of specimens from two sources: participants in routine county health examinations in selected counties of Norway, and Red Cross donors (Jellum et al. 1995). Blood samples were obtained from approximately 264,000 health examination donors recruited from Finnmark, Oslo, Sogn og Fjordane, Oppland (*n* = 87,647; enrolled between 1974 and 1977), and other areas in Norway (*n* = 176,881; recruited between 1985 and 1991). Age at entry was between 20 and 49 years, with the majority of subjects enrolled between 35 and 49 years of age. Approximately 29,000 Red Cross donors, 20–65 years of age at entry and primarily from the Oslo region, were enrolled between 1972 and 1989. Serum from all participants was separated and stored at –25°C.

Cases and controls were selected among Janus cohort members with the following characteristics: no prior history of cancer (except nonmelanoma skin cancer) at baseline blood collection; baseline blood collection between 1972 and 1978; and at least 0.8 mL of stored serum. TGCT cases diagnosed between enrollment through 31 December 1999 were identified though linkage with the Norwegian Cancer Registry, using the Norwegian population identification number. Of the 61 identified cases, two were excluded from further analysis on the basis of pathology (one case of spermato cytic seminoma, a rare subtype of TGCT arising among older adults, and one case of testicular mesothelioma).

One male control was matched to each case by region, time period of blood draw (1-year strata), and age group at blood draw (2-year strata). Controls were required to be cancer-free during the blood draw-to-diagnosis window of the respective case (specifically, within the same 2-year stratum starting from the midpoint of the case’s blood draw stratum). To increase statistical power, we also included four additional controls from another project within the Janus cohort that was assayed in parallel with the TGCT samples at the same laboratory. These four controls met the aforementioned matching criteria for—and were in the same assay batch as—four cases.

The serum samples of 10 cases and 12 controls were not successfully analyzed because of laboratory equipment malfunction or technician error, leaving 49 case–control pairs (49 cases, 51 controls) with organochlorine measurements available for analysis.

Demographic and other data were obtained from the Norwegian Cancer Registry and census, as well as from the Janus cohort database containing information from the original health examinations and other surveys. In particular, information on height and body mass index (BMI) at the time of blood collection was available for the subset of subjects who had entered the Janus cohort as a result of their participation in routine county health examinations (34 cases, 37 controls).

### Laboratory analyses

Concentrations of 11 organochlorine pesticides, their metabolites, or related chemicals [β-hexachlorocyclohexane (β-HCH), dieldrin, γ-HCH, HCB, mirex, *o,p*′-DDT, *p,p*′-DDE, *p,p*′-DDT, heptachlor epoxide, oxychlordane, *trans*-nonachlor] and 34 PCB congeners (PCBs 18, 28, 44, 49, 52, 66, 74, 87, 99, 101, 118, 128, 138, 146, 149, 151, 153, 156, 157, 167, 170, 172, 177, 178, 180, 183, 187, 189, 194, 195, 196, 201, 206, 209; International Union of Pure and Applied Chemistry) and lipid levels were measured at the Centers for Disease Control and Prevention, National Center for Environmental Health in 0.7 mL serum, using previously published methods (Engel et al. 2007). In brief, serum samples were first spiked with isotopically labeled internal standards, then purified via automated accelerated solvent extraction and high-resolution gel permeation chromatography on a high-performance liquid chromatograph. Concentrated extracts were analyzed by gas chromatography/ high-resolution mass spectrometry. Selected PCB congeners and organochlorine pesticides in each sample were quantified from ^13^C isotope dilution continuing calibration plots, which automatically corrected for extraction efficiency. Values below the instrumental limits of detection (LOD) were imputed using a parametric model-based estimation procedure (Lubin et al. 2004). Using measurements among controls for a target analyte, we used maximum likelihood methods to estimate parameters for the log-normal distribution. For each measurement < LOD, we randomly sampled a value from the appropriate log-normal distribution as the imputed value. As a quality control check for possible errors in measurement due to interfering compounds, the ratio of ^35^Cl to ^37^Cl was calculated for each analyte and compared with the expected ratio for that analyte. Analyte measurements with observed ion ratios (IRs) outside ± 20% of the expected IR were flagged as being out of tolerance and were recoded based on their proximity to the LOD; flagged values within 10 times the instrumental LOD were treated as if they were < LOD and imputed as described above, while flagged values greater than 10 times the instrumental LOD were recoded to missing data.

Total lipid concentration was calculated for each subject using measurements of total cholesterol and triglycerides in an additional 0.1 mL serum (Phillips et al. 1989). Masked quality control samples, including single samples from a large pool and pairs of replicate samples, were interspersed among study samples to assess intrabatch and interbatch variability. Case–control matched pairs were included in the same batch.

### Statistical analysis

All measures were adjusted for total lipids (Phillips et al. 1989). Analytes with measurements < LOD or flagged as out of tolerance in at least half the subjects (PCBs 87, 149, and 151) were excluded from further analysis. Analyses were conducted for individual analytes, total chlordanes (heptachlor epoxide, oxychlordane, *trans*-nonachlor), total PCBs, and *a priori* groupings of PCB congeners based on degree of chlorination, predicted enzyme induction, and estrogenicity (McFarland and Clarke 1989; Wolff et al. 1997). Concentrations of total chlordanes and PCB groupings were calculated by summing the concentrations of all relevant analytes (including those < LOD). For the calculation of groupings, analyte missing values for cases and controls were imputed from control measurement data using the previously described imputation procedure, with the exception that the LOD was specified as the lower limit for imputed values.

Intrabatch coefficients of variation (CV) were < 10 for most analytes (median 7; range, 4–21; see Supplemental Material, Table 1, available online (doi:10.1289/ehp.0800359. S1 via http://dx.doi.org/). Interbatch CVs were considerably larger (median 37; range, 17–165). To assess the impact of possibly problematic assay batches on our results, we reviewed the values of samples from a quality control (QC) pool, one sample of which was included in every batch. For each analyte, we identified batches for which the QC pool measurement was extreme (± 2 SDs from the QC pool mean across all batches) and reran our analyses with these batches removed. When we reanalyzed our data excluding such batches, our findings did not change.

The DDT metabolites were highly correlated, with Spearman correlation coefficients ≥ 0.7, as were the three chlordane compounds. Two distinct clusters of strong correlation were observed among PCBs, consisting of congeners 28–66 and 138–209.

We conducted statistical analyses using SAS, version 9.1 (SAS Institute Inc., Cary, NC, USA). All tests were two-sided. Comparisons in lipid-adjusted organochlorine levels between matched case–control pairs were performed using the Wilcoxon signed-rank test. Conditional logistic regression modeling was performed to calculate odds ratios (ORs) and 95% confidence intervals (CIs) relating categories of lipid-adjusted analyte levels with risk of TGCT. The analyte levels were categorized into tertiles based on the levels in controls, with the lowest tertile used as the reference category. Findings from analyses of dichotomized analyte levels, using control medians as cut points, yielded similar findings and are not presented here. Analyses were also performed to investigate associations with semi-nomas (*n* = 34), the major histologic subtype of TGCTs observed in our study. Analyses restricted to non seminomas (*n* = 8) and tumors of mixed histology (*n* = 5) were not performed because of the small numbers of such cases.

We performed additional analyses among the subset of individuals for whom information on height and BMI at blood collection was available (34 cases, 37 controls) to assess whether adjustment for these variables (categorized using control tertiles as cut points) as model covariates led to changes in observed associations with OC compounds.

## Results

The mean age at blood draw among TGCT cases and controls was 33.7 years (range, 20–48) and 33.9 years (21–48), respectively. The mean age at diagnosis for cases was 44.3 years (27–62), with an average length of follow-up to diagnosis of 10.7 years (1–27). Thirty-four of the 49 cases were seminomas, 8 were nonseminomas, 5 were of mixed histology, and 2 were of unknown histology.

Results for case–control comparisons of lipid-adjusted concentrations of OC insecticide compounds are summarized in Table 1. We observed suggestive evidence of an association with *p,p*′-DDE (*p*_Wilcoxon_ = 0.07). Two chlordane metabolites were also associated with increased risk of TGCT at a borderline level of statistical significance: oxychlordane and *trans*-nonachlor (*p*_Wilcoxon_ = 0.05 and 0.07, respectively). No association with heptachlor epoxide was observed (*p*_Wilcoxon_ = 0.18). The summed concentration of all three chlordane compounds was significantly greater among cases than controls (*p*_Wilcoxon_ = 0.048). The other OC insecticide compounds (*o,p*′-DDT, *p,p*′-DDT, β-HCH, dieldrin, γ-HCH, HCB, and mirex) were not found to be associated with TGCT (*p*_Wilcoxon_ = 0.10).

Results for analyses of PCBs are summarized in Table 2. The summed concentration of all PCB congeners was not associated with TGCT (*p*_Wilcoxon_ = 0.17), although associations were observed for PCBs 99, 138, 167, and 183 (*p*_Wilcoxon_ = 0.04, 0.06, 0.02 and 0.07, respectively. Results for all congeners are available in Supplemental Material, Table 2 (doi:10.1289/ ehp.0800359.S1). Analyses of most PCB groupings were null, although borderline statistically significant associations were observed for congeners with a mixed-type pattern of cytochrome p450 induction (*p*_Wilcoxon_= 0.07).

Findings from analyses investigating associations with seminoma are summarized in Table 3 [results for all compounds are available in Supplemental Material, Table 2 (doi:10.1289/ehp.0800359.S1)]. We observed evidence of association with *p,p*′-DDE and oxychlordane (*p*_Wilcoxon_ = 0.06 and 0.006, respectively), but not with *trans*-nonachlor or total chlordanes. Although no association with total PCBs was observed, several congeners were associated with seminoma. Compared with controls, seminomas had significantly lower concentrations of PCBs 44, 49, and 52 (*p*_Wilcoxon_ = 0.003, 0.03, and 0.03, respectively) and significantly higher concentrations of PCBs 99, 138, 153, 167, 183, and 195 (*p*_Wilcoxon_ = 0.01, 0.02, 0.04, 0.008, 0.01, and 0.04, respectively). Four PCB groupings were associated with an increased risk of seminoma [moderate chlorination, *p*_Wilcoxon_ = 0.04; mixed-type induction, *p*_Wilcoxon_ = 0.03; Wolff 2 and 2B groupings (Wolff et al. 1997), *p*_Wilcoxon_ = 0.05 and 0.04, respectively], whereas the Wolff 1A grouping was associated with reduced risk (*p*_Wilcoxon_ = 0.004). We did not perform analyses restricted to cases of nonseminoma or mixed histology because of the small number of cases.

Our findings did not meaningfully change when we repeated our analyses excluding three cases diagnosed within the first 2 years of follow-up (results not shown). The observed associations with OC compounds remained upon additional model adjustment for BMI and height within the subset of individuals for whom these covariate data were available.

## Discussion

In this nested case–control investigation of OC compounds and TGCT risk, we observed elevated prediagnostic serum concentrations oxychlordane, *trans*-nonachlor, and *p,p*- ′ DDE among TGCT cases compared with controls. We also observed significantly higher levels of PCBs 99, 138, 153, 167, 183, and 195 among seminoma cases versus controls as well as significantly lower levels than controls of PCBs 44, 49, and 52.

Chlordane is a cyclodiene insecticide commonly used in agriculture and termite control from 1945 until around 1970, at which time countries began banning or restricting its use. (Norway instituted a ban in 1968.) Our finding that TGCT cases had higher levels of oxychlordane and *trans-*nonachlor than controls offers further support to previously published evidence suggesting that chlordanes affect the risk of TGCT development. We observed a particularly strong and statistically significant association with oxychlordane for seminoma cases. In the STEED study, statistically significant associations with TGCT were observed for *cis*-nonachlor and *trans*-nonachlor, whereas oxychlordane was associated with seminoma risk (McGlynn et al. 2008). A significant association with *cis*-non-achlor was also observed in the Swedish study (Hardell et al. 2003). In addition, the case mothers in that study had significantly higher levels of *cis*-nonachlor, *trans*-nonachlor, and the sum of chlordanes compared with control mothers, suggesting that the antenatal period may be a particularly important time window of exposure for influencing TGCT development. No associations with chlordane congeners were observed in the Washington State study (Biggs et al. 2008). The biologic basis for a chlordane effect on TGCT development is unclear. High doses of chlordane have been reported to have deleterious effects on testicular tissues, with reductions observed in testis weight, seminiferous tubule diameter, and spermatogenesis (Al-Omar et al. 2000; Balash et al. 1987). Findings from *in vitro* studies suggest that the influence of chlordane on sex hormone signaling may be complex. There is evidence that chlordane is a weak agonist for human estrogen receptors α and β (Kojima et al. 2004; Lemaire et al. 2006) and a weak antagonist for the human androgen receptor (Kojima et al. 2004; Lemaire et al. 2004). However, chlordane may also exert antiestrogenic effects by inhibiting aromatase activity through estrogen-related receptor α1 antagonism (Yang and Chen 1999).

*p,p*′-DDE is the primary metabolite of the first modern synthesized insecticide, DDT. DDT was widely used in agriculture and vector control in the mid- to late 20th century until concerns regarding its environmental persistence and possible health effects led several developed countries to ban its use in the 1970s and 1980s (including Norway, in 1970). Our observed association with *p,p*′-DDE is consistent with findings from the STEED study, the largest and only other prospective study of OC compounds and TGCTs (McGlynn et al. 2008). No associations with this compound were observed in the case–control studies conducted in Washington State (Biggs et al. 2008) and Sweden (Hardell et al. 2003), although the latter study, with its small size, was underpowered to detect associations of moderate magnitude. Research suggests that high exposure to *p,p*′-DDE may increase TGCT risk through the induction of antiandrogenic effects, as this compound has been shown to be a competitive antagonist for the androgen receptor (Danzo 1997; Kelce et al. 1995; Xu et al. 2006).

Considerable interest has focused on the possible health effects of PCBs, a class of OC compounds used widely in the manufacturing of electrical equipment and other industrial applications until the institution of bans in the 1970s (banned in Norway in 1980). Experimental evidence suggests that some PCBs may exert estrogenic and possibly anti-androgenic effects and induce cytochrome p450 activity (Bonefeld-Jorgensen et al. 2001; DeCastro et al. 2006; Portigal et al. 2002; Soto et al. 1995; Wolff et al. 1997). In our study, we did not observe a clear association between PCB levels and TGCT risk. However, seminoma cases had significantly elevated concentrations of PCBs 99, 138, 153, 167, 183, and 195 compared with controls. We also observed that seminoma cases had significantly lower levels of PCBs 44, 49, and 52 than controls. The associations with semi-noma risk observed for the moderate chlorination, mixed-type induction, and Wolff 1A, 2 and 2B groupings appear to reflect the associations with the aforementioned congeners.

We are not aware of any clear biologic basis for the observed differences in the direction of association with seminoma risk for these two groups. In fact, members of each group (PCBs 99, 138, 153, 183, 44, 49, and 52) have demonstrated similar biologic effects, namely, phenobarbital-type cytochrome p450 induction activity and estrogenicity. (Bonefeld-Jorgensen et al. 2001; DeCastro et al. 2006; Wolff et al. 1997). The only other published findings regarding PCBs and TGCTs come from the Swedish case–control study (Hardell et al. 2003, 2004); in that study, no difference in PCB levels between cases and controls was observed. In maternal comparisons, however, mothers of seminoma and nonseminoma cases had significantly higher levels of total PCBs and several individual congeners, including PCBs 99, 138, 153, 183, and 195 (PCB 167 was not measured). Maternal levels of PCB 52 were not found to be associated with reduced cancer risk (PCBs 44 and 49 were not measured). In summary, the published epidemiologic evidence, although limited, suggests that some PCB congeners may be associated with TGCT risk.

An important strength of this study is the use of serum samples collected prior to diagnosis, thus eliminating the possibility that our measurements were affected by the disease state. Given the rarity and young age at onset of TGCTs, most cohort studies are not feasible for investigations of this cancer. The Janus cohort is one of the few cohorts in the world with a large enough number of subjects recruited as young adults and with stored serum to make prospective serologic investigations of TGCTs feasible. Moreover, the time period during which the samples were collected (1972–1978) is particularly relevant for studying the health effects of OC compounds, as these chemicals were still in use or had only recently been banned at the time of collection.

The most notable limitation of this study is its small sample size, which greatly limited the statistical power to detect associations of moderate magnitude. As a consequence, we cannot rule out the existence of modest effects on TGCT risk from among the other investigated OC compounds. In addition, given the large number of measured compounds and generally borderline significance level of our observed findings, we cannot rule out the possibility that some of the observed associations may have arisen due to chance, although it is worth noting that associations with oxychlordane, *trans*-nonachlor, and *p,p*′-DDE have been previously reported. Moreover, some of the observed associations may have resulted from the moderate to strong correlations between many compounds. Finally, we were unable to investigate the relative importance of specific time windows of exposure to OC compounds, most notably the antenatal and early childhood periods, on TGCT risk.

The mean age at diagnosis and proportion of cases with seminoma histology in this study were higher than those observed in population-based cancer registries. This is likely a reflection of the older age distribution of cohort participants at baseline relative to the general population. Nonetheless, the seminoma findings should be interpreted with caution, in light of their small sample size and the fact that they were not the primary end point of this study.

In conclusion, this study provides additional but qualified epidemiologic evidence that serum levels of *p,p*′-DDE, oxychlordane, and *trans*-nonachlor compounds—and more speculatively, some PCB congeners (44 and 167 in particular)—may be associated with risk of TGCTs, including seminomas. These chemicals, although long banned in developed countries, are still a concern because of their continued presence in certain food sources (Fattore et al. 2006; Freijer et al. 2001; Schecter et al. 2001) and the continued use of OC pesticides in developing countries (Gorman and Tynan 2003; Mathur 1993; Santilo et al. 1997). Additional investigations into the relationship between TGCTs and exposure to OC compounds, particularly in early life, are warranted.

## Correction

Many values in the original version published online were incorrect, especially in the tables. Also, “oxychlordane” was incorrectly called “oxychlor” in two places in the “Discussion.” These errors have been corrected here.

## Figures and Tables

**Table 1:** Serum concentration of selected organochlorine insecticide compounds and risk of TGCTs.

	Lipid-adjusted analyte concentration (ng/g lipid)			ORs for concentration tertiles (reference group: tertile 1)				
	Cases (*n* = 49)	Controls (*n* = 51)		Tertile 1	Tertile 2		Tertile 3	
Analyte	Median (range)	Median (range)	*p*_Wilcoxon_^a^	*n*_Co_/*n*_Ca_	*n*_Co_/*n*_Ca_	OR (95% CI)^b^	*n*_Co_/*n*_Ca_	OR (95% CI)
DDT metabolite								
*o,p*′-DDT	20.7 (6.0–220.5)	16.6 (0.3–171.9)	0.36	17/16	17/11	0.8 (0.2–2.6)	17/21	1.4 (0.4–4.5)
*p,p*′-DDT	226.0 (92.2–584.1)	194.6 (29.3–661.0)	0.23	17/11	17/20	2.3 (0.7–7.2)	17/18	2.1 (0.6–7.2)
*p,p*′-DDE	2098.5 (750.0–9512.2)	1833.2 (224.9–7436.3)	0.07	17/11	17/18	1.8 (0.6–5.5)	17/20	2.2 (0.7–6.5)

Chlordane								
Heptachlor epoxide	12.0 (0.1–43.6)	11.9 (0.2–27.4)	0.18	16/12	16/16	1.7 (0.5–5.8)	16/20	2.4 (0.6–9.1)
Oxychlordane	13.4 (0.6–77.4)	11.8 (0.8–79.4)	0.05	16/11	15/20	3.0 (0.8–11.1)	15/18	3.2 (0.6–16.8)
*trans*-Nonachlor	23.2 (10.7–76.9)	20.5 (8.3–99.2)	0.07	17/10	17/21	3.1 (0.8–11.6)	17/18	2.6 (0.7–8.9)
Total	47.3 (17.9–143.2)	45.2 (19.0–206.0)	0.048	17/13	17/17	1.8 (0.5–6.5)	17/19	2.3 (0.6–7.2)

Other insecticide								
β-HCH	143.0 (58.4–414.0)	129.7 (59.7–295.9)	0.20	17/15	17/13	1.0 (0.4–2.7)	17/21	1.8 (0.5–6.1)
Dieldrin	54.2 (0.6–177.9)	54.7 (0.7–251.0)	0.46	17/11	17/22	3.3 (0.8–12.7)	17/15	2.1 (0.5–9.5)
γ-HCH	6.4 (0.1–42.4)	6.8 (0.2–45.1)	0.89	17/17	17/15	1.0 (0.3–3.1)	17/17	1.1 (0.2–5.0)
HCB	713.4 (199.5–2458.2)	568.6 (168.6–3185.9)	0.26	17/15	17/13	1.1 (0.3–3.7)	17/21	2.9 (0.5–15.2)
Mirex	1.7 (0.1–26.0)	1.5 (0.1–14.9)	0.10	17/16	16/13	0.9 (0.3–2.9)	16/17	1.2 (0.4–3.0)

Abbreviations: *n*_Ca_, number of cases; *n*_Co_, number of controls.

a *p*-Value from Wilcoxon signed-rank test of matched case–control sets.

b ORs and 95% CIs calculated from conditional logistic regression of matched case–control sets.

**Table 2:** Serum concentration of selected PCB congeners and PCB groupings and risk of TGCTs.

	Lipid-adjusted analyte concentration (ng/g lipid)			ORs for concentration tertiles (reference group: tertile 1)				
	Cases (*n* = 49)	Controls (*n* = 51)		Tertile 1	Tertile 2		Tertile 3	
Analyte	Median (range)	Median (Range)	*p*_Wilcoxon_^a^	*n*_Co_/*n*_Ca_	*n*_Co_/*n*_Ca_	OR (95% CI)^b^	*n*_Co_/*n*_Ca_	OR (95% CI)
Selected PCB congener^c^								
44	16.4 (0.5–92.4)	16.9 (0.6–108.3)	0.14	17/18	16/12	0.4 (0.1–2.5)	17/18	0.6 (0.1–3.8)
49	9.8 (0.4–81.7)	10.9 (0.2–79.2)	0.41	17/16	17/13	0.7 (0.1–3.4)	17/20	1.2 (0.2–7.6)
52	24.4 (3.7–103.4)	23.2 (5.3–116.9)	0.49	17/18	17/10	0.6 (0.2–1.9)	17/20	1.0 (0.3–3.5)
99	68.5 (35.0–408.4)	62.6 (24.2–183.4)	0.04	17/9	17/19	2.2 (0.7–7.3)	17/21	2.2 (0.8–5.9)
138	199.6 (87.2–891.7)	177.3 (79.9–394.5)	0.06	17/15	17/10	0.7 (0.3–2.0)	17/24	1.8 (0.6–5.1)
153	431.5 (166.0–1922.5)	399.3 (187.1–860.7)	0.10	17/16	17/14	0.9 (0.3–2.5)	17/19	1.2 (0.4–3.4)
167	11.2 (0.6–45.0)	9.1 (0.1–24.3)	0.02	16/10	16/13	2.2 (0.5–9.2)	16/19	4.4 (1.0–19.8)
183	30.4 (13.2–151.5)	27.6 (11.1–59.0)	0.07	17/14	16/15	1.0 (0.4–2.6)	16/18	1.3 (0.5–3.5)
195	7.9 (0.2–43.4)	6.9 (0.3–17.6)	0.19	16/10	16/20	1.9 (0.7–5.0)	15/15	1.7 (0.6–4.6)
Total PCBs^d^	1845.4 (1014.8–8542.7)	1751.1 (800.5–3470.0)	0.17	17/14	17/16	1.1 (0.5–2.7)	17/19	1.3 (0.5–3.8)

Degree of chlorination^e^								
Low	308.1 (56.9–1753.9)	291.2 (70.4–1335.5)	0.89	17/13	17/17	1.7 (0.5–6.5)	17/19	2.2 (0.4–10.9)
Moderate	1348.7 (596.8–6955.4)	1295.2 (557.8–2866.2)	0.10	17/13	17/16	1.2 (0.5–2.8)	17/20	1.5 (0.6–4.1)
High	87.5 (35.6–722.2)	83.7 (31.6–202.1)	0.42	17/15	17/11	0.7 (0.3–1.8)	17/23	1.4 (0.6–3.3)

Mixed-function oxidase induction^f^								
Phenobarbital type	918.2 (412.2–4786.9)	880.5 (404.0–1876.6)	0.16	17/12	17/18	1.3 (0.6–3.0)	17/19	1.5 (0.5–4.1)
Mixed type	451.0 (218.6–2112.1)	426.7 (170.7–919.1)	0.07	17/13	17/14	1.1 (0.4–2.7)	17/22	1.7 (0.6–4.8)

Wolff group^g^								
1	172.9 (113.3–846.1)	166.2 (70.6–450.5)	0.22	17/13	17/18	1.4 (0.5–3.9)	17/18	1.4 (0.5–3.9)
1A	51.0 (6.7–275.8)	53.6 (8.8–304.4)	0.32	17/18	17/10	0.4 (0.1–1.7)	17/21	1.0 (0.2–4.4)
1B	118.9 (49.8–742.3)	109.4 (42.0–332.9)	0.15	17/10	17/19	1.8 (0.7–5.0)	17/20	1.8 (0.7–4.8)
2	531.2 (278.0–2458.0)	493.9 (210.4–1021.6)	0.10	17/13	17/13	1.0 (0.4–2.3)	17/23	1.6 (0.6–4.1)
2A	229.7 (115.8–1037.9)	203.8 (89.3–603.7)	0.16	17/13	17/14	1.4 (0.4–4.3)	17/22	1.9 (0.6–5.6)
2B	297.3 (130.3–1420.1)	275.8 (121.1–581.6)	0.10	17/15	17/13	0.9 (0.4–2.2)	17/21	1.4 (0.5–3.7)
3	784.2 (312.5–3985.0)	738.8 (333.9–1599.8)	0.13	17/13	17/18	1.3 (0.5–3.1)	17/18	1.3 (0.5–3.8)

Abbreviations: *n*_Ca_, number of cases; *n*_Co_, number of controls.

a *p*-Value from Wilcoxon signed-rank test of matched case–control sets.

b ORs and 95% CIs calculated from conditional logistic regression of matched case–control sets.

c Congeners significantly associated with TGCTs or seminoma (Table 3) reported. Results for all congeners are provided in Supplemental Material, Table 2 (doi:10.1289/ehp.0800359.S1).

d Sum of all 31 congeners included in the analysis.

e Low: PCBs 11, 18, 28, 44, 49, 52, 66, and 74; moderate: PCBs 99, 105, 110, 118, 128, 138, 146, 153, 156, 157, 167, 170, 172, 177, 178, 180, 183, 187, and 189; high: PCBs 194, 195, 196, 201, 206, and 209.

f Phenobarbital-type induction: PCBs 11, 52, 66, 99, 146, 153, 180, 183, 194, 195, 196, 206, and 209; mixed-type induction: PCBs 105, 118, 128, 138, 156, 157, 167, 170, and 189.

g Wolff 1A: PCBs 44, 49, and 52; Wolff 1B: PCBs 177, 187, and 201; Wolff 2A: PCBs 66, 74, 105, 118, 156, and 167; Wolff 2B: PCBs 128, 138, and 170; Wolff 3: PCBs 99, 153, 180, 183, and 196.

**Table 3:** Serum concentration of selected organochlorine compounds and risk of seminoma tumors.

	Median lipid-adjusted analyte concentration (ng/g lipid)			ORs for concentration tertiles (reference group: tertile 1)^a^				
				Tertile 1	Tertile 2		Tertile 3	
Analyte	Seminoma (*n* = 34)	Controls (*n* = 34)	*p*_Wilcoxon_^b^	*n*_Co_/*n*_Ca_	*n*_Co_/*n*_Ca_	OR (95% CI)^c^	*n*_Co_/*n*_Ca_	OR (95% CI)
Selected organochlorine insecticide								
*p,p*′-DDE	2100.4	1768.2	0.06	12/10	13/10	1.0 (0.3–3.8)	9/14	2.2 (0.5–8.7)
Oxychlordane	14.3	11.8	0.006	10/6	13/15	3.2 (0.6–15.7)	9/13	5.1 (0.7–36.8)
*trans*-Nonachlor	22.6	19.6	0.21	13/9	10/15	3.7 (0.7–19.0)	11/10	1.6 (0.4–6.0)
Total chlordanes	45.4	45.2	0.13	11/9	12/12	1.4 (0.3–5.5)	10/12	1.6 (0.4–6.6)

PCB								
Total PCBs^d^	1819.7	1856.6	0.12	10/9	11/11	1.1 (0.4–3.3)	13/14	1.2 (0.4–4.1)

Selected PCB congener^e^								
44	15.0	17.0	0.003	9/13	9/9	0.3 (0.03–3.0)	15/12	0.2 (0.01–2.0)
49	9.5	11.6	0.03	10/12	10/8	0.3 (0.02–3.0)	14/14	0.3 (0.02–4.7)
52	23.1	26.8	0.03	10/14	10/6	0.3 (0.05–1.6)	14/14	0.4 (0.07–2.3)
99	75.4	63.5	0.01	12/5	10/12	5.6 (0.9–36.3)	12/17	4.4 (1.0–20.5)
138	203.3	177.2	0.02	11/9	12/8	0.9 (0.3–3.0)	11/17	2.1 (0.6–7.2)
153	439.3	395.9	0.04	12/10	9/11	1.4 (0.4–4.7)	13/13	1.2 (0.4–4.3)
167	11.9	8.9	0.008	11/6	12/8	2.3 (0.4–13.4)	10/15	6.7 (1.1–42.9)
183	31.9	27.4	0.01	10/8	14/11	1.0 (0.3–3.5)	8/14	2.9 (0.6–13.7)
195	8.3	7.1	0.04	9/6	13/13	2.0 (0.5–7.2)	8/13	3.0 (0.8–11.7)

Degree of chlorination^f^								
Low	309.9	291.6	0.46	11/10	8/11	2.3 (0.4–14.1)	15/13	0.5 (0.04–5.4)
Moderate	1421.8	1289.6	0.04	10/8	12/11	1.2 (0.4–3.4)	12/14	1.5 (0.4–5.4)
High	98.5	85.0	0.28	11/11	13/6	0.4 (0.1–1.5)	10/17	1.9 (0.6–5.9)

Mixed-function oxidase induction^g^								
Phenobarbital type	935.1	882.0	0.10	11/8	11/12	1.4 (0.5–3.8)	12/14	1.6 (0.5–5.8)
Mixed type	465.4	424.4	0.03	11/8	11/10	1.3 (0.4–3.9)	12/16	2.0 (0.6–6.9)

Wolff group^h^								
1	172.2	178.8	0.53	10/11	11/11	0.9 (0.2–3.3)	13/12	0.8 (0.2–3.1)
1A	49.3	54.0	0.004	10/14	9/7	0.3 (0.1–1.8)	15/13	0.3 (0.04–1.9)
1B	128.4	111.8	0.08	11/8	11/9	1.0 (0.3–3.3)	12/17	1.9 (0.6–6.0)
2	551.3	497.3	0.05	11/8	11/8	1.0 (0.3–3.5)	12/18	1.9 (0.6–5.9)
2A	243.4	207.0	0.12	12/10	10/7	1.0 (0.2–3.9)	12/17	1.6 (0.5–5.4)
2B	303.7	273.6	0.04	11/8	12/10	1.1 (0.4–3.2)	11/16	2.4 (0.6–8.8)
3	791.8	719.0	0.07	12/8	11/13	1.6 (0.5–4.7)	11/13	1.8 (0.5–6.6)

Abbreviations: *n*_Ca_, number of cases; *n*_Co_, number of controls.

a Category cut points are the same as those used in Tables 1 and 2.

b *p*-Value from Wilcoxon signed-rank test of matched case–control sets.

c ORs and 95% CIs calculated from conditional logistic regression of matched case–control sets.

d Sum of all 31 congeners included in the analysis.

e Results for all analytes are provided in Supplemental Material, Table 3 (doi:10.1289/ehp.0800359.S1).

f Low: PCBs 11, 18, 28, 44, 49, 52, 66, and 74; moderate: PCBs 99, 105, 110, 118, 128, 138, 146, 153, 156, 157, 167, 170, 172, 177, 178, 180, 183, 187, and 189; high: PCBs 194, 195, 196, 201, 206, and 209.

g Phenobarbital-type induction: PCBs 11, 52, 66, 99, 146, 153, 180, 183, 194, 195, 196, 206, and 209; mixed-type induction: PCBs 105, 118, 128, 138, 156, 157, 167, 170, and 189.

h Wolff 1A: PCBs 44, 49, and 52; Wolff 1B: PCBs 177, 187, and 201; Wolff 2A: PCBs 66, 74, 105, 118, 156, and 167; Wolff 2B: PCBs 128, 138, and 170; Wolff 3: PCBs 99, 153, 180, 183, and 196 (Wolff et al. 1997).
